# 4-Bromo-*N*-cyclo­hexyl­benzene­sulfonamide

**DOI:** 10.1107/S1600536810026796

**Published:** 2010-07-10

**Authors:** Peter John, Faiza Anwar, Islam Ullah Khan, Shahzad Sharif, Edward R. T. Tiekink

**Affiliations:** a Materials Chemistry Laboratory, Department of Chemistry, Government College University, Lahore 54000, Pakistan; b Department of Chemistry, University of Malaya, 50603 Kuala Lumpur, Malaysia

## Abstract

The title compound, C_12_H_16_BrNO_2_S, adopts an L-shaped conformation with the central C—S—N—C torsion angle being −77.8 (3)°. The crystal packing features N—H⋯O hydrogen bonds, which lead to *C*(4) chains propagating in [010]; the second O atom is involved in short intra­molecular C—H⋯O contacts.

## Related literature

For related structures and background information on sulfon­amides, see: Khan *et al.* (2010 ▶); Sharif *et al.* (2010 ▶).
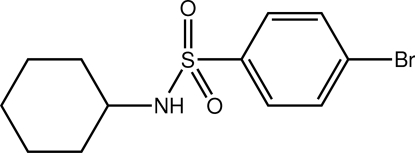


## Experimental

### 

#### Crystal data

C_12_H_16_BrNO_2_S
*M* *_r_* = 318.24
Monoclinic, 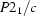

*a* = 11.2539 (5) Å
*b* = 6.2575 (3) Å
*c* = 19.9743 (10) Å
β = 97.214 (3)°
*V* = 1395.48 (11) Å^3^
*Z* = 4
Mo *K*α radiation
μ = 3.09 mm^−1^
*T* = 293 K
0.24 × 0.12 × 0.08 mm

#### Data collection

Bruker APEXII CCD diffractometer
Absorption correction: multi-scan (*SADABS*; Sheldrick, 1996 ▶) *T*_min_ = 0.218, *T*_max_ = 0.529
12505 measured reflections
3199 independent reflections
1620 reflections with *I* > 2σ(*I*)
*R*_int_ = 0.048

#### Refinement

*R*[*F* ^2^ > 2σ(*F* ^2^)] = 0.048
*wR*(*F* ^2^) = 0.132
*S* = 1.01
3199 reflections
157 parameters
1 restraint
H atoms treated by a mixture of independent and constrained refinement
Δρ_max_ = 0.52 e Å^−3^
Δρ_min_ = −0.46 e Å^−3^

### 

Data collection: *APEX2* (Bruker, 2007 ▶); cell refinement: *SAINT* (Bruker, 2007 ▶); data reduction: *SAINT* ; program(s) used to solve structure: *SHELXS97* (Sheldrick, 2008 ▶); program(s) used to refine structure: *SHELXL97* (Sheldrick, 2008 ▶); molecular graphics: *ORTEP-3* (Farrugia, 1997 ▶) and *DIAMOND* (Brandenburg, 2006 ▶); software used to prepare material for publication: *publCIF* (Westrip, 2010 ▶).

## Supplementary Material

Crystal structure: contains datablocks global, I. DOI: 10.1107/S1600536810026796/hb5542sup1.cif

Structure factors: contains datablocks I. DOI: 10.1107/S1600536810026796/hb5542Isup2.hkl

Additional supplementary materials:  crystallographic information; 3D view; checkCIF report

## Figures and Tables

**Table 1:** Hydrogen bonds and short intramolecular contacts (Å, °)

*D*—H⋯*A*	*D*—H	H⋯*A*	*D*⋯*A*	*D*—H⋯*A*
C2—H2⋯O2	0.93	2.53	2.903 (4)	104
C7—H7⋯O2	0.98	2.54	2.992 (4)	108
N1—H1n⋯O1^i^	0.88 (3)	2.03 (3)	2.898 (4)	169 (3)

Symmetry code: (i) 
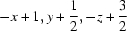
.

**Fig. 1.**
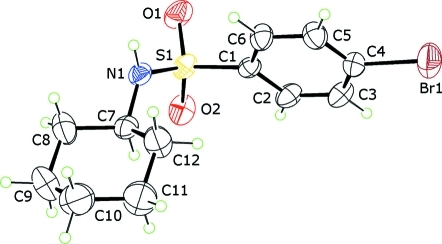
The molecular structure of (I) showing displacement ellipsoids at the 35% probability level.

**Fig. 2.**
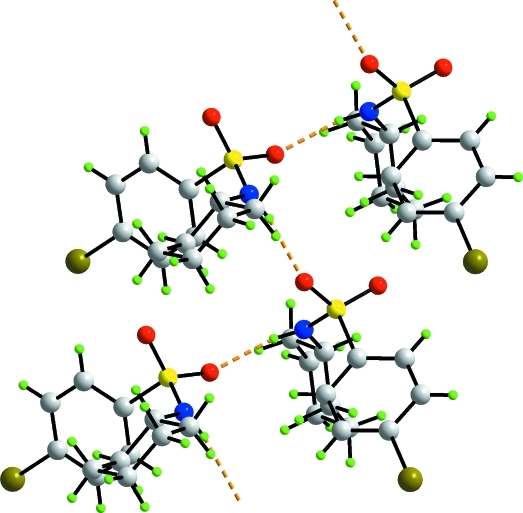
A view of the supramolecular chain along the b axis in (I) mediated by N–H···O hydrogen bonding (orange dashed lines) in (I). Colour code: Br, olive; S, yellow; O, red; N, blue; C, grey; and H, green.

## supplementary crystallographic information

### Comment

The title sulfonamide has been prepared as a part of on-going structural studies of such compounds (Khan *et al.*, 2010; Sharif *et al.*, 2010).

Overall, the molecule in (I), Fig. 1, has an *L*-shaped conformation. This is best quantified in the C1–S1–N1–C7 torsion angle of -77.8 (3) °. When viewed down the spine of the benzene ring, the cyclohexyl group, with a regular chain conformation, appears almost side-on. With respect to the plane through the benzene ring, the O2 atom is roughly co-planar [the C2–C1–S1–O2 torsion angle is 18.8 (4) °]. By contrast, the O1 and N1 atoms lie to either side [C2–C1–S1–O1 = -109.1 (3) ° and C2–C11–S1–N1 = 136.8 (3) °]. This conformation allows for the formation of two intramolecular C–H···O2 short contacts and it is not surprising that the O2 atom does not participate in significant intermolecular interactions. Supramolecular chains along the *b* axis are found in the crystal structure. These are mediated by N–H···O1 hydrogen bonding, Fig. 2 and Table 1.

### Experimental

To 4-bromobenzene sulfonylchloride (499 mg, 1.96 mmol) in distilled water (10 ml), was added cyclohexylamine (225 ml, 1.96 mmol) with continuous stirring at room temperature. The pH of the reaction mixture was maintained at 8 using a 3% sodium carbonate solution. The progress of the reaction was monitored by TLC. After the consumption of all the reactants, the precipitates were filtered, dried and crystallized using ethyl acetate to yield colourless prisms of (I), m.pt. 375 K.

### Refinement

The C-bound H atoms were geometrically placed (C–H = 0.93–0.98 Å) and refined as riding with *U_iso_*(H) = 1.2*U_eq_*(C). The N-bound H atom was refined with the distance restraint N–H = 0.88±0.01 Å, and with *U_iso_*(H) = 1.2*U_eq_*(N). In the final refinement two low angle reflections evidently effected by the beam stop were omitted, *i.e*. 1 0 0 and 0 0 2.

### Crystal data

**Table:**

C_12_H_16_BrNO_2_S	*F*(000) = 648
*M**_r_* = 318.24	*D*_x_ = 1.515 Mg m^−^^3^
Monoclinic, *P*2_1_/*c*	Mo *K*α radiation, λ = 0.71073 Å
Hall symbol: -P 2ybc	Cell parameters from 2185 reflections
*a* = 11.2539 (5) Å	θ = 2.6–20.1°
*b* = 6.2575 (3) Å	µ = 3.09 mm^−^^1^
*c* = 19.9743 (10) Å	*T* = 293 K
β = 97.214 (3)°	Prism, colourless
*V* = 1395.48 (11) Å^3^	0.24 × 0.12 × 0.08 mm
*Z* = 4	

### Data collection

**Table:**

Bruker APEXII CCD diffractometer	3199 independent reflections
Radiation source: fine-focus sealed tube	1620 reflections with *I* > 2σ(*I*)
graphite	*R*_int_ = 0.048
φ and ω scans	θ_max_ = 27.5°, θ_min_ = 1.8°
Absorption correction: multi-scan (*SADABS*; Sheldrick, 1996)	*h* = −14→14
*T*_min_ = 0.218, *T*_max_ = 0.529	*k* = −7→8
12505 measured reflections	*l* = −25→23

### Refinement

**Table:**

Refinement on *F*^2^	Primary atom site location: structure-invariant direct methods
Least-squares matrix: full	Secondary atom site location: difference Fourier map
*R*[*F*^2^ > 2σ(*F*^2^)] = 0.048	Hydrogen site location: inferred from neighbouring sites
*wR*(*F*^2^) = 0.132	H atoms treated by a mixture of independent and constrained refinement
*S* = 1.01	*w* = 1/[σ^2^(*F*_o_^2^) + (0.0552*P*)^2^ + 0.4435*P*] where *P* = (*F*_o_^2^ + 2*F*_c_^2^)/3
3199 reflections	(Δ/σ)_max_ < 0.001
157 parameters	Δρ_max_ = 0.52 e Å^−^^3^
1 restraint	Δρ_min_ = −0.46 e Å^−^^3^

### Special details

**Table:**

Geometry. All s.u.'s (except the s.u. in the dihedral angle between two l.s. planes) are estimated using the full covariance matrix. The cell s.u.'s are taken into account individually in the estimation of s.u.'s in distances, angles and torsion angles; correlations between s.u.'s in cell parameters are only used when they are defined by crystal symmetry. An approximate (isotropic) treatment of cell s.u.'s is used for estimating s.u.'s involving l.s. planes.
Refinement. Refinement of *F*^2^ against ALL reflections. The weighted *R*-factor *wR* and goodness of fit *S* are based on *F*^2^, conventional *R*-factors *R* are based on *F*, with *F* set to zero for negative *F*^2^. The threshold expression of *F*^2^ > 2σ(*F*^2^) is used only for calculating *R*-factors(gt) *etc*. and is not relevant to the choice of reflections for refinement. *R*-factors based on *F*^2^ are statistically about twice as large as those based on *F*, and *R*- factors based on ALL data will be even larger.

### Fractional atomic coordinates and isotropic or equivalent isotropic displacement parameters (Å^2^)

**Table:**

	*x*	*y*	*z*	*U*_iso_*/*U*_eq_	
Br1	0.03064 (5)	1.13493 (9)	0.90414 (3)	0.1022 (3)	
S1	0.32555 (8)	0.51051 (14)	0.73331 (5)	0.0523 (3)	
O1	0.4205 (2)	0.4370 (4)	0.78229 (16)	0.0763 (9)	
O2	0.2437 (2)	0.3587 (3)	0.70013 (15)	0.0685 (8)	
N1	0.3875 (3)	0.6368 (4)	0.67782 (17)	0.0497 (8)	
H1N	0.450 (2)	0.714 (5)	0.6936 (17)	0.060*	
C1	0.2414 (3)	0.6900 (5)	0.77706 (17)	0.0435 (8)	
C2	0.1289 (3)	0.6312 (6)	0.7906 (2)	0.0584 (10)	
H2	0.0958	0.5019	0.7746	0.070*	
C3	0.0658 (3)	0.7644 (7)	0.8278 (2)	0.0660 (11)	
H3	−0.0102	0.7259	0.8372	0.079*	
C4	0.1155 (3)	0.9538 (6)	0.8508 (2)	0.0582 (10)	
C5	0.2265 (4)	1.0149 (6)	0.8369 (2)	0.0597 (10)	
H5	0.2586	1.1454	0.8524	0.072*	
C6	0.2899 (3)	0.8824 (6)	0.8000 (2)	0.0555 (10)	
H6	0.3655	0.9223	0.7904	0.067*	
C7	0.3239 (3)	0.7049 (5)	0.61271 (19)	0.0515 (9)	
H7	0.2584	0.6039	0.6000	0.062*	
C8	0.4105 (5)	0.6910 (9)	0.5601 (3)	0.0944 (16)	
H8A	0.4799	0.7797	0.5739	0.113*	
H8B	0.4376	0.5446	0.5569	0.113*	
C9	0.3511 (6)	0.7641 (13)	0.4915 (3)	0.121 (2)	
H9A	0.4100	0.7639	0.4599	0.146*	
H9B	0.2886	0.6634	0.4751	0.146*	
C10	0.2986 (5)	0.9797 (11)	0.4935 (3)	0.1082 (19)	
H10A	0.2572	1.0147	0.4493	0.130*	
H10B	0.3623	1.0833	0.5043	0.130*	
C11	0.2126 (5)	0.9940 (9)	0.5448 (3)	0.1004 (17)	
H11A	0.1436	0.9042	0.5310	0.121*	
H11B	0.1848	1.1402	0.5474	0.121*	
C12	0.2716 (4)	0.9235 (7)	0.6137 (2)	0.0770 (13)	
H12A	0.3344	1.0241	0.6297	0.092*	
H12B	0.2126	0.9260	0.6452	0.092*	

### Atomic displacement parameters (Å^2^)

**Table:**

	*U*^11^	*U*^22^	*U*^33^	*U*^12^	*U*^13^	*U*^23^
Br1	0.0808 (4)	0.1222 (5)	0.1086 (5)	0.0104 (3)	0.0315 (3)	−0.0430 (3)
S1	0.0472 (5)	0.0401 (5)	0.0703 (7)	0.0070 (4)	0.0101 (5)	0.0059 (5)
O1	0.0616 (17)	0.0779 (19)	0.088 (2)	0.0278 (15)	0.0053 (16)	0.0236 (16)
O2	0.0717 (18)	0.0390 (14)	0.098 (2)	−0.0085 (13)	0.0227 (17)	−0.0093 (13)
N1	0.0382 (16)	0.0525 (18)	0.058 (2)	−0.0045 (13)	0.0052 (15)	−0.0051 (15)
C1	0.0385 (19)	0.0442 (19)	0.047 (2)	0.0044 (16)	0.0015 (16)	0.0099 (16)
C2	0.043 (2)	0.052 (2)	0.079 (3)	−0.0051 (18)	0.003 (2)	−0.003 (2)
C3	0.040 (2)	0.076 (3)	0.083 (3)	0.001 (2)	0.010 (2)	0.001 (2)
C4	0.052 (2)	0.070 (3)	0.053 (3)	0.012 (2)	0.0077 (19)	−0.006 (2)
C5	0.065 (2)	0.058 (2)	0.058 (3)	−0.007 (2)	0.014 (2)	−0.007 (2)
C6	0.050 (2)	0.057 (2)	0.062 (3)	−0.0101 (18)	0.0186 (19)	−0.0018 (19)
C7	0.044 (2)	0.051 (2)	0.059 (3)	−0.0072 (17)	0.0042 (19)	−0.0070 (18)
C8	0.098 (4)	0.127 (4)	0.062 (3)	0.026 (3)	0.024 (3)	−0.016 (3)
C9	0.118 (5)	0.192 (7)	0.057 (4)	0.009 (5)	0.024 (3)	−0.018 (4)
C10	0.083 (4)	0.158 (6)	0.084 (4)	−0.013 (4)	0.012 (3)	0.046 (4)
C11	0.104 (4)	0.113 (4)	0.085 (4)	0.023 (3)	0.015 (3)	0.034 (3)
C12	0.095 (3)	0.071 (3)	0.066 (3)	0.021 (2)	0.016 (3)	0.008 (2)

### Geometric parameters (Å, °)

**Table:**

Br1—C4	1.893 (4)	C7—C12	1.490 (5)
S1—O2	1.427 (3)	C7—C8	1.523 (5)
S1—O1	1.431 (3)	C7—H7	0.9800
S1—N1	1.591 (3)	C8—C9	1.518 (8)
S1—C1	1.769 (3)	C8—H8A	0.9700
N1—C7	1.467 (5)	C8—H8B	0.9700
N1—H1N	0.88 (3)	C9—C10	1.475 (8)
C1—C6	1.376 (5)	C9—H9A	0.9700
C1—C2	1.378 (5)	C9—H9B	0.9700
C2—C3	1.372 (5)	C10—C11	1.499 (7)
C2—H2	0.9300	C10—H10A	0.9700
C3—C4	1.366 (6)	C10—H10B	0.9700
C3—H3	0.9300	C11—C12	1.517 (6)
C4—C5	1.369 (5)	C11—H11A	0.9700
C5—C6	1.368 (5)	C11—H11B	0.9700
C5—H5	0.9300	C12—H12A	0.9700
C6—H6	0.9300	C12—H12B	0.9700
			
O2—S1—O1	119.11 (17)	C8—C7—H7	108.1
O2—S1—N1	108.71 (17)	C9—C8—C7	111.0 (4)
O1—S1—N1	106.31 (17)	C9—C8—H8A	109.4
O2—S1—C1	107.33 (16)	C7—C8—H8A	109.4
O1—S1—C1	105.45 (17)	C9—C8—H8B	109.4
N1—S1—C1	109.69 (15)	C7—C8—H8B	109.4
C7—N1—S1	123.6 (2)	H8A—C8—H8B	108.0
C7—N1—H1N	116 (2)	C10—C9—C8	112.5 (5)
S1—N1—H1N	115 (2)	C10—C9—H9A	109.1
C6—C1—C2	120.3 (3)	C8—C9—H9A	109.1
C6—C1—S1	120.4 (3)	C10—C9—H9B	109.1
C2—C1—S1	119.3 (3)	C8—C9—H9B	109.1
C3—C2—C1	119.7 (3)	H9A—C9—H9B	107.8
C3—C2—H2	120.2	C9—C10—C11	111.6 (5)
C1—C2—H2	120.2	C9—C10—H10A	109.3
C2—C3—C4	119.5 (4)	C11—C10—H10A	109.3
C2—C3—H3	120.3	C9—C10—H10B	109.3
C4—C3—H3	120.3	C11—C10—H10B	109.3
C5—C4—C3	121.3 (4)	H10A—C10—H10B	108.0
C5—C4—Br1	119.0 (3)	C10—C11—C12	110.9 (4)
C3—C4—Br1	119.7 (3)	C10—C11—H11A	109.5
C6—C5—C4	119.4 (4)	C12—C11—H11A	109.5
C6—C5—H5	120.3	C10—C11—H11B	109.5
C4—C5—H5	120.3	C12—C11—H11B	109.5
C5—C6—C1	119.9 (3)	H11A—C11—H11B	108.0
C5—C6—H6	120.1	C7—C12—C11	112.5 (4)
C1—C6—H6	120.1	C7—C12—H12A	109.1
N1—C7—C12	113.8 (3)	C11—C12—H12A	109.1
N1—C7—C8	108.2 (3)	C7—C12—H12B	109.1
C12—C7—C8	110.5 (4)	C11—C12—H12B	109.1
N1—C7—H7	108.1	H12A—C12—H12B	107.8
C12—C7—H7	108.1		
			
O2—S1—N1—C7	39.2 (3)	Br1—C4—C5—C6	178.2 (3)
O1—S1—N1—C7	168.6 (3)	C4—C5—C6—C1	0.3 (6)
C1—S1—N1—C7	−77.8 (3)	C2—C1—C6—C5	0.6 (6)
O2—S1—C1—C6	−164.1 (3)	S1—C1—C6—C5	−176.5 (3)
O1—S1—C1—C6	68.0 (3)	S1—N1—C7—C12	91.1 (4)
N1—S1—C1—C6	−46.1 (3)	S1—N1—C7—C8	−145.7 (3)
O2—S1—C1—C2	18.8 (4)	N1—C7—C8—C9	−178.8 (4)
O1—S1—C1—C2	−109.1 (3)	C12—C7—C8—C9	−53.5 (6)
N1—S1—C1—C2	136.8 (3)	C7—C8—C9—C10	54.4 (7)
C6—C1—C2—C3	−0.8 (6)	C8—C9—C10—C11	−55.0 (7)
S1—C1—C2—C3	176.3 (3)	C9—C10—C11—C12	54.5 (7)
C1—C2—C3—C4	0.1 (6)	N1—C7—C12—C11	176.7 (4)
C2—C3—C4—C5	0.8 (7)	C8—C7—C12—C11	54.8 (5)
C2—C3—C4—Br1	−178.4 (3)	C10—C11—C12—C7	−55.3 (6)
C3—C4—C5—C6	−1.0 (6)		

### Hydrogen-bond geometry (Å, °)

**Table:**

*D*—H···*A*	*D*—H	H···*A*	*D*···*A*	*D*—H···*A*
C2—H2···O2	0.93	2.53	2.903 (4)	104
C7—H7···O2	0.98	2.54	2.992 (4)	108
N1—H1n···O1^i^	0.88 (3)	2.03 (3)	2.898 (4)	169 (3)

Symmetry codes:  (i) −*x*+1, *y*+1/2, −*z*+3/2.
